# TrAmplification of Human Dental Follicle Cells by *piggyBac* Transposon - Mediated Reversible Immortalization System

**DOI:** 10.1371/journal.pone.0130937

**Published:** 2015-07-14

**Authors:** Yan Wu, Ge Feng, Jinlin Song, Yuanyuan Zhang, Yong Yu, Lan Huang, Leilei Zheng, Feng Deng

**Affiliations:** 1 Department of Orthodontics, Stomatological Hospital of Chongqing Medical University, Chongqing, P. R. China; 2 Chongqing Key Laboratory of Oral Disease and Biomedical Sciences, Chongqing, P. R. China; 3 Chongqing Municipal Key Laboratory of Oral Biomedical Engineering of Higher Education, Chongqing, P. R. China; 4 Wake Forest Institute for Regenerative Medicine (WFIRM), Wake Forest University Health Sciences, Winston-Salem, North Carolina, United States of America; The Roslin Institute, UNITED KINGDOM

## Abstract

Dental follicle cells (DFCs) are the precursor cells of periodontium. Under certain differentiation conditions, DFCs can be induced to differentiate into chondrogenic, osteogenic and adipogenic cells. However, DFCs has limited lifespan in vitro, so it’s difficult to harvest enough cells for basic research and translational application. pMPH86 is a *piggyBac transposon*-mediated vector which contains SV40 T-Ag cassette that can be removed by flippase recognition target (FRT) recombinase. Here we demonstrated the pMPH86 can effectively amplify human DFCs through reversible immortalization. The immortalized DFCs (iDFCs) exhibit higher proliferate activity, which can be reversed to its original level before immortalization when deimmortalized by FLP recombinase. The iDFCs and deimmortalized DFCs (dDFCs) express most DFC markers and maintain multiple differentiation potential in vitro as they can be induced by BMP9 to differentiate into chondrogenic, osteogenic and adipogenic cells evidenced by gene expression and protein marker. We also proved telomerase activity of iDFCs are significantly increased and maintained at a high level, while the telomerase activity of primary DFCs was relatively low and decreased with every passage. After SV40 T-Ag was removed to deimmortalize the cells, telomerase activity was reduced to its original level before immortalization and decreased with passages just the same as primary DFCs. These results suggest that *piggyBac* immortalization system could be a potential strategy to amplify primary cells, which is critical for regenerative research and further clinical application.

## Introduction

DFCs are the progenitor cells of osteoblasts, periodontal ligament cells (PDLCs) and cementoblasts within dental follicle [1–3]. These cells can respectively develop into components of periodontal tissues, such as periodontal ligament fibers, cementum and alveolar bone when dental tissue injure happens. Under certain differentiation conditions, DFCs can also be induced into chondrogenic, osteogenic, adipogenic and neuronal cells [4–7] and form new bone [8,9], periodontal tissue and dentin–pulp complex [4,10,11,12] *in vivo*. The latest research manifested that as the progenitor cells of periodontal ligament cells (PDLCs), DFCs are more advantageous for periodontal tissue regeneration than PDLCs or other dental-derived cells [4]. Moreover, human DFCs could be isolated from the discarded wisdom tooth, which makes them the ideal cell source for periodontal tissue regeneration and other potential application in regenerative medicine.

One major challenge is to harvest enough DFCs from limited tissue and to expand sufficient amount of cells for basic research and clinical application. Stem cells usually lost their original characters due to over passages. Development of technology to generate a multitude of DFCs without losing their stemness is highly desirable. Therapeutic application of DFCs would demand even more cells, which requires *in vitro* expansion post-harvest. One solution to this problem is to reversibly or conditionally immortalize DFCs safely and effectively. The major way to immortalize cells is overexpression of oncogene or inhibition of the tumor suppressor genes. [13]. SV40 T-Ag is one of the widely used genes for immortalization [14–17]. The efficiency to immortalize primary cells by employing retroviral vector to overexpress SV40 T-Ag was comparatively low, largely because the viral titters of retrovirus was low when long gene fragment is transducted.[18–23]. Thus, how to transfer the immortalizing elements into the objective cells with high efficiency is the major obstacle to efficient immortalization.

*PiggyBac* (PB) transposon is a mobile genetic element and it is one of the most favorable non-viral gene delivery tools [24–27]. It can efficiently transposes between vectors and chromosomes. Traditional DNA transposons vector includes one plasmid which expresses the transposase and another plasmid encoding target genes. The *piggyBac*-mediated vector pMPH86 contains SV40 T-Ag gene with FRT sites located on both ends of it and hygromycin B for selection, and it has been used before to immortalize mouse embryonic fibroblasts, but the biological property of the deimmortalized cells hasn’t been fully tested.[28].

In this study, we determined whether the *piggyBac* vector pMPH86 can effectively amplify human DFCs through reversible immortalization system. The infection efficiency was compared with retroviral vector-mediated system. And also cell proliferation rate, telomerase activity and multi-potent differentiation potential of DFCs, iDFCs and dDFCs were thoroughly investigated.

## Material and Methods

### Isolation of human dental follicle cells

The study is approved by the Ethics Committee of Chongqing Medical University and performed with written informed consent of the patients. Embedded human third molars with immature developing roots (ie, roots developed to <2/3 their full size) were obtained from three young adults (18 to 20 years old). Dental follicles were washed with PBS(including 100 mg/ml streptomycin (Gibco) and 100 units/ml penicillin) and digested in 1% collagenase Isolution(Sigma) for 40 min at 37°C and then 0.25% trypsin (Gibco) for another 5 min. The digested tissue were suspended in 5 ml DMEM/F12 1:1 complete medium (HyClone) (including 10% fetal bovine serum (Gibco)). Then the mixture of digested tissue and single cells were transferred into a 75 cm^2 culture^ flask (Corning) and incubated at 37°C with 5% CO_2_. The culture medium was added to 10 ml after 24 h and changed every 3–4 days. 7–10 days later, the cells were collected and prepared for limiting dilution procedure to obtain single-colony-derived strains as previously illustrated [29]. The cell suspensions were diluted such that each well of the 96-well plate was seeded with approximately 1 cell. One colony of each dental follicle was collected, cultured, and passaged at ratio at 1:2 when they reached 80% confluence. The experiments were carried out by using cells at passage 3.

### *PiggyBac* Mediated immortalized HDFCs

To set up immortalized DFCs (iDFCs), DFCs at passage (*p3*) were transducted with *piggyBac* vector pMPH86(Gifts from Dr. Tong-Chuan He), and *piggyBac* transposase expression adenoviral vector AdpBase (Gifts from Dr. Tong-Chuan He). Hygromycin B was used for selection for one week to establish stable iDFC pools. Deimmortalized dental follicle cells (dDFCs) were generated by infecting iDFCs with AdFLP, which can effectively recognize the FLP sit and cut SV40 T-Ag out. Aliquots of DFCs, iDFCs and dDFCs were frozened in liquid nitrogen tank.

### Retroviral vector-mediated immortalized HDFCs

Retrovirus *SSR#69*, which also expresses SV40 T Ag, was used to transfect DFCs at passage (*p3*) as previously reported [17]. Hygromycin B was used for selection for one week to establish stable iDFC pools.

### Recombinant adenoviruses

AdEasy technology was used to generate recombinant adenoviruses to transfect BMP9, PPARγ2, FLP, GFP and RFP gene into the cells as previously demonstrated [30–33]. The first step is to amplify the coding gene of BMP9, mouse PPARγ2, FLP recombinase, GFP and RFP and then the amplified products were cloned into an adenoviral vector. At last, HEK293 cells were used to produce recombinant adenoviruses. and the generated adenoviruses were named AdPPARγ2 which also express RFP as a marker. AdBMP9 and AdFLP express GFP as a marker. AdRFP and AdGFP were used as negative controls [34,35].

### Cell Proliferation Assay

To detect cell proliferation of DFCs, iDFCs and dDFCs, cell counting kit-8 (CCK-8,Dojindo) was used. Cells were transfered into 96-well plate (5 *10^2^ cells/well). The culture medium was discarded at certain time points, and 100 μl fresh culture medium plus 10 μl CCK-8 solution were injected into each well. The plate were incubated in the dark for 2 hours, and the absorbance at 450 nm was measured using a microplate reader (Thermo). Each experiment was performed using five wells per experimental condition, and the experiment lasted for ten continuous days. The assays were done in triplicate.

The same amount of DFCs, iDFCs and dDFCs were seeded at 10% confluence. Cells were digested with trypsin at specified time points and then counted. Each experiment was performed using five wells per experimental condition, and the experiment lasted for ten continuous days. The assays were done in triplicate.

### Immunofluorescence Microscopy

To identify cell surface markers of DFCs, iDFCs and dDFCs, cells were first transferred into a 24-well plate and cultured for 14 hours. Cells were then fixed with 4% polyoxymethylene(PFA) for 30 min, followed by perforation with 0.5% TritonX-100 for 20 min, and block with 10% BSA for 1 hour. First antibodies used were vimentin antibody (ImmunoWay, YT4880, 1:2000) and CK14 antibody (Santa Cruz Biotechnology, SC23878, 1:1000). After incubation with first antibody for 14hours, cells were rinsed with PBS and then incubated with DyLight 488 AffiniPure Goat AntiRabbit IgG or DyLight 488 AffiniPure Goat Anti-Mouse IgG respectively for half an hour. Cell nuclei were stained with 100 ng/ml DAPI for 5 min. Then photos of stained cells were taken under a confocal laser scanning microscopy.

### Telomerase activity analysis

To test the stemness of DFCs, iDFCs and dDFCs, telomeric repeat amplification protocol (TRAP) was used to test the telomerase activity of the cells according to the manufacturer’s protocol (TeloTAGGG Telomerase PCR ELISA^PLUS^, Roche, USA). First of all, telomerase in the cells add telomeric repeats (TTAGGG) to the 3’-end of the P1-TS-primer. The resulting products are then amplified by PCR with the Internal standard included in the same container. The obtained PCR products are divided into two vessels, followed by denaturation and hybridization with digoxigenin-labeled probes. Then the products are checked with an antibody against digoxigenin and the peroxidase substrate TMB. Cell extract treated under 85°C for 10 min were used as negative controls.

### *In Vitro* Multi-differentiation of DFCs, iDFCs and dDFCs

DFCs, iDFCs and dDFCs were seeded into a six-well plate (1 *10^5^ cells/well) separately and further cultured overnight for eight hours.

To induce osteogenic differentiation, cells were infected with AdBMP9, and the medium was replaced with StemPro Osteogenesis Differentiation Kit (Gibco) for two weeks. Then the differentiated cells were fixed with 4% polyoxymethylene for 20 min, followed by Alkaline phosphatase (Beyotime) staining and Alizarin Red (Beyotime) staining to assess mineral deposition. Cells infected with AdGFP were used as control.

To induce chondrogenic differentiation, cells were infected with AdBMP9, and the medium was replaced with Chondrogesis Differentiation medium (Biowit) for two weeks. The induced cells were fixed with 4% polyoxymethylene for 20 min, followed by alcian blue staining. Cells infected with AdGFP were used as control.

To induce adipogenic differentiation, cells were infected with Ad PPARγ2, and the medium was replaced with Adipogesis Differentiation medium (Biowit) for one week. The differentiated cells were fixed with 4% polyoxymethylene for 20 min and then stained with 0.3% Oil Red O (Sigma) solution to evaluate adipogenesis. Cells infected with AdRFP were used as control.

### Alkaline phosphatase (ALP) activity assay

To quantitatively assess the ALP activity of the osteogenic differentiated cells, Alkaline Phosphatase Assay Kit (Nanjing Jiancheng Bioengineering Institute) was used according to the manufacture’s instruction. The absorbance at indicated time points was measured at 520 nm via a 96-well plate reader (Thermo). Each assay was performed in three wells and the results were repeated in triplicate.

### Quantitative Real-Time Polymerase Chain Reaction (qRT-PCR)

To detect multi-potency differentiation of DFCs, iDFCs and dDFCs, total RNA of cells was isolated by MiniBEST Universal RNA Extraction Kit (Takara) and reverse transcription were performed using PrimeScript RT reagent Kit with gDNA Eraser (Takara) to produce complementary deoxyribonucleic acid (cDNA). qRT-PCR was done by the CFX Connect Real-Time PCR Detection System (Bio-Rad). Mean fold changes of gene expression relative to glyceraldehyde 3-phosphate dehydrogenase (GAPDH) were calculated using the 2-^ΔΔCt^ methods.

### Flow Cytometry Analysis

DFCs, iDFCs and dDFCs were digested and then resuspended in PBS containing 10%FBS to a final concentration of 1*10^7^ cells/ml. Distribute 100 μl aliquots of the cell suspension into each tube. Add antibodies to cells and incubate for 20 min in the dark. Antibodies used were FITC-conjugated mouse anti- human CD34 (BD Biosciences, 555821; 20 μl), phycoerythrin(PE)-conjugated mouse anti-human CD73 (BD Biosciences, 550257; 20 μl), FITC-conjugated mouse anti-human CD105 (BD Biosciences, 561443; 5 μl) and FITC-conjugated anti-human Strol-1. Finally, flow cytometry(BD Biosciences) was used to analyze the stained cells.

### Western blotting analysis

To assess osteogenic, chondrogenic and adipogenic related protein expression, Western blotting was performed as described [34,36]. Samples were normalized for protein concentration. Nitrocellulose membranes were probed with primary antibodies including anti-SOX9 antibody (Abcam,ab185230,1:2000), anti-Osteopontin antibody (Abcam,ab8448, 1:1000), anti- PPARγ2 antibody(Abcam, ab45036,1:1000) or anti-GAPDH antibody (Abcam, ab181602,1:10000). And then horseradish peroxidase-conjugated secondary antibody was used to incubate the nitrocellulose membranes for one hour. The proteins of interest were examined by using BeyoECL Plus kit (Beyotime).

### Statistical Analysis

All quantitative experiments were done in three independent experiment and the results were determined by three independent experiment. Data were showed as mean±SD. Statistical significance was determined by student’s t test and a value of *p* < 0.05 was considered statistically significant.

## Results

### Single-colony-derived strains of primary DFCs could be formed with limiting dilution method

**Fig 1:**
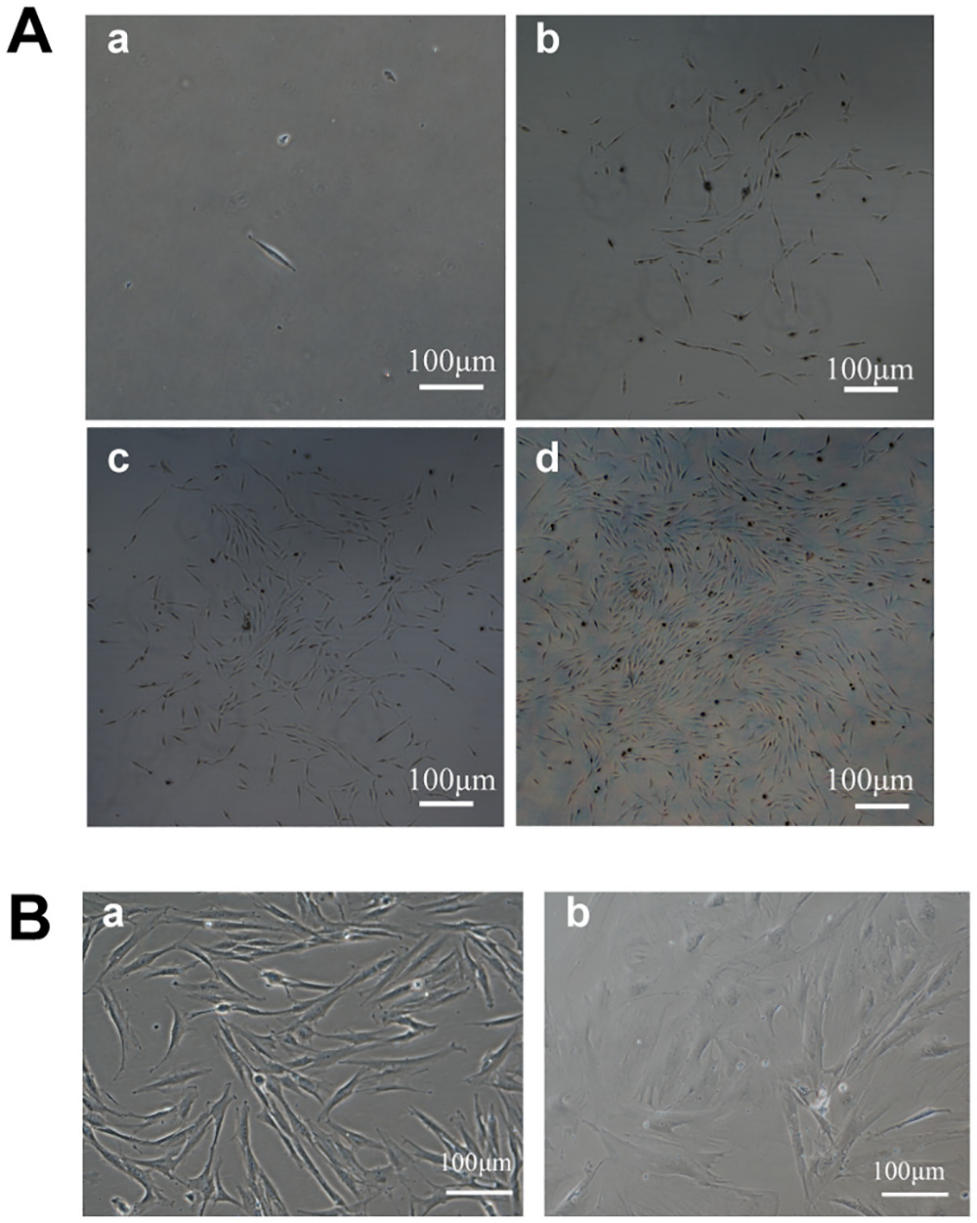
Establishment of single-colony-derived DFCs. (A). Clone forming of DFCs. Limiting dilution was used for single clone selection. One single cell was observed after the procedure (a), several days later the cells formed a clone in which more than 50 cells were observed (d). (B). Morphological change of DFCs after several passages. (a). At passage 3, DFCs were fibroblast-like cells. (b). Cells became flat after more than 5 passages.

Single-colony-derived strains of primary DFCs were formed with limiting dilution method (Fig 1A), and the DFCs displayed fibroblast-like with a small size of cytoplasmic (Fig 1B, panel a). The growth rate of DFCs was relatively stable up to about five passages. After that, the cells lost their typical mesenchymal stem cell morphology (Fig 1B, panel a) and became flat (Fig 1B, panel b), while the growth rate became even slower.

### The *piggyBac* Transposon system can effectively immortalize DFCs with high efficiency

**Fig 2:**
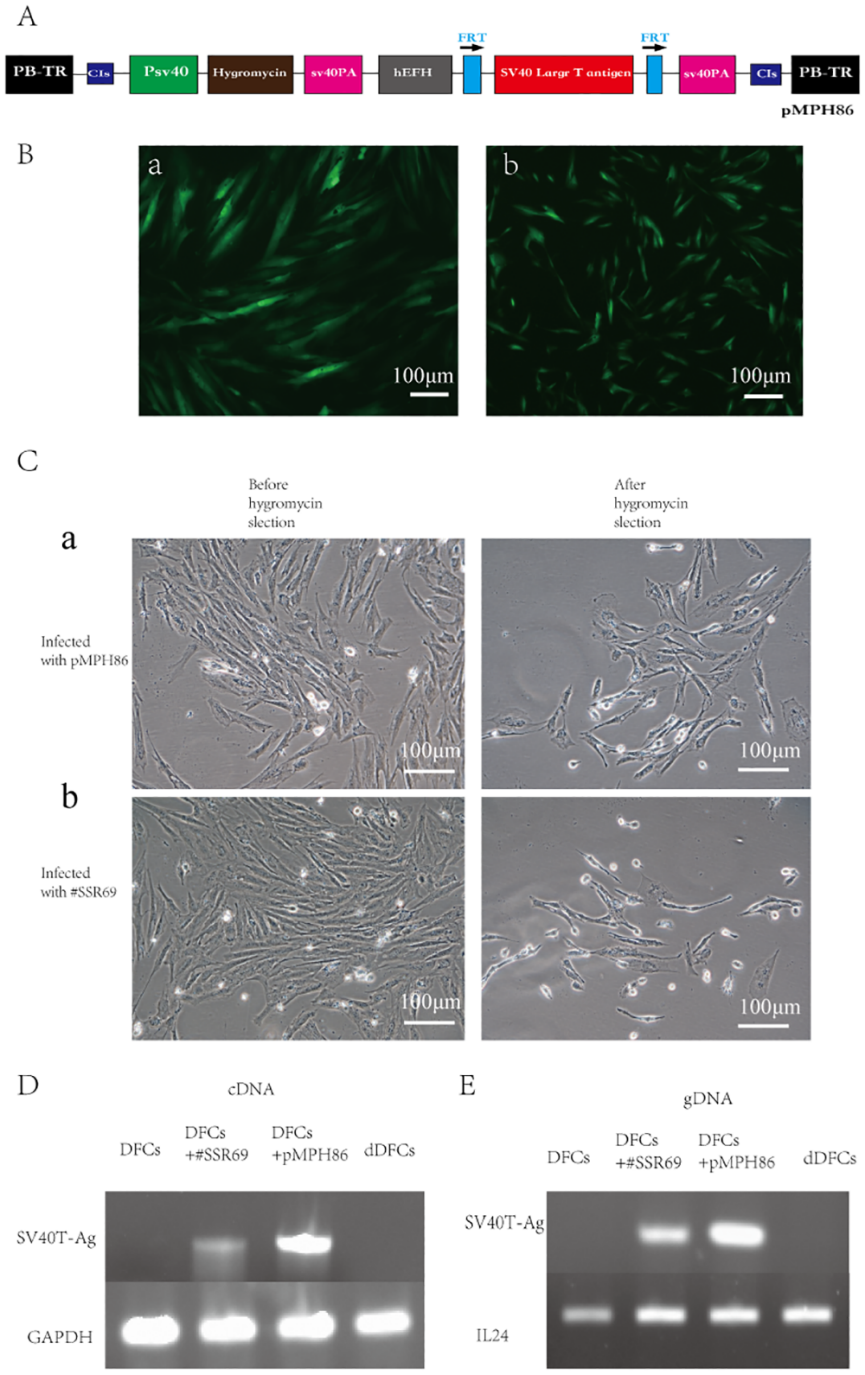
Infection efficiency of *piggyBac* Transposon system. (A). Schematic diagram of pMPH86, the *piggyBac* Transposon-mediated reversible vector for immortalization, that includes SV40 T-Ag and hygromycin B expression cassette. (B). Cell transducted by AdpBase or AdFLP for 24 hours. (a). DFCs transducted efficiently by adenoviral vectors AdpBase to establish immortalized dental follicle cells (iDFCs) (b). Efficient transduction of iDFCs by adenoviral vectors AdFLP to get deimmortalized dental follicle cells (dDFCs). (C). Hygromycin B selection. (a). Survival rate of cells infected with pMPH86 is 30%. (b). Survival rate of cells infected with *SSR#69* is 10%. (D). Expression of SV40T-Ag in cells infected with pMPH86 or *SSR#69*. (E). Integration of SV40T-Ag gene in cells infected with pMPH86 or *SSR#69*.

The generated *piggyBac* vector, named pMPH86, contains hygromycin expression cassette and SV40 T-Ag expression site with FRT sites on both sides (Fig 2A).

Two strains of immortalized DFCs were obtained by infecting cells with pMPH86 or *SSR#69*. The dental follicle cells transducted by pMPH86 were named immortalized dental follicle cells (iDFCs). And the deimmortalized DFCs were established by removing the immortalizing gene SV40 large T antigen from iDFCs using FLP recombinase. The infection efficiency could be assessed by hygromycin B selection. The survival rate of cells infected with pMPH86 is 30%, while that of cells infected with *SSR#69* is only 10%(Fig 2C).

We also extract cDNA and gDNA from these two kinds of immortalized cells, and the PCR results showed cells infected with pMPH86 exhibit higher integration rate of SV40 T-Ag (Fig 2D) and higher expression of SV40T-Ag compared with cells infected with *SSR#69* (Fig 2E). After deimmortalization, there was no expression of SV40T-Ag and the gene had been removed from genomic DNA completely (Fig 2D and 2E).

### The *piggyBac* Transposon-mediated iDFCs exhibit high proliferative activity, which can be reversed by deimmortalization

**Fig 3:**
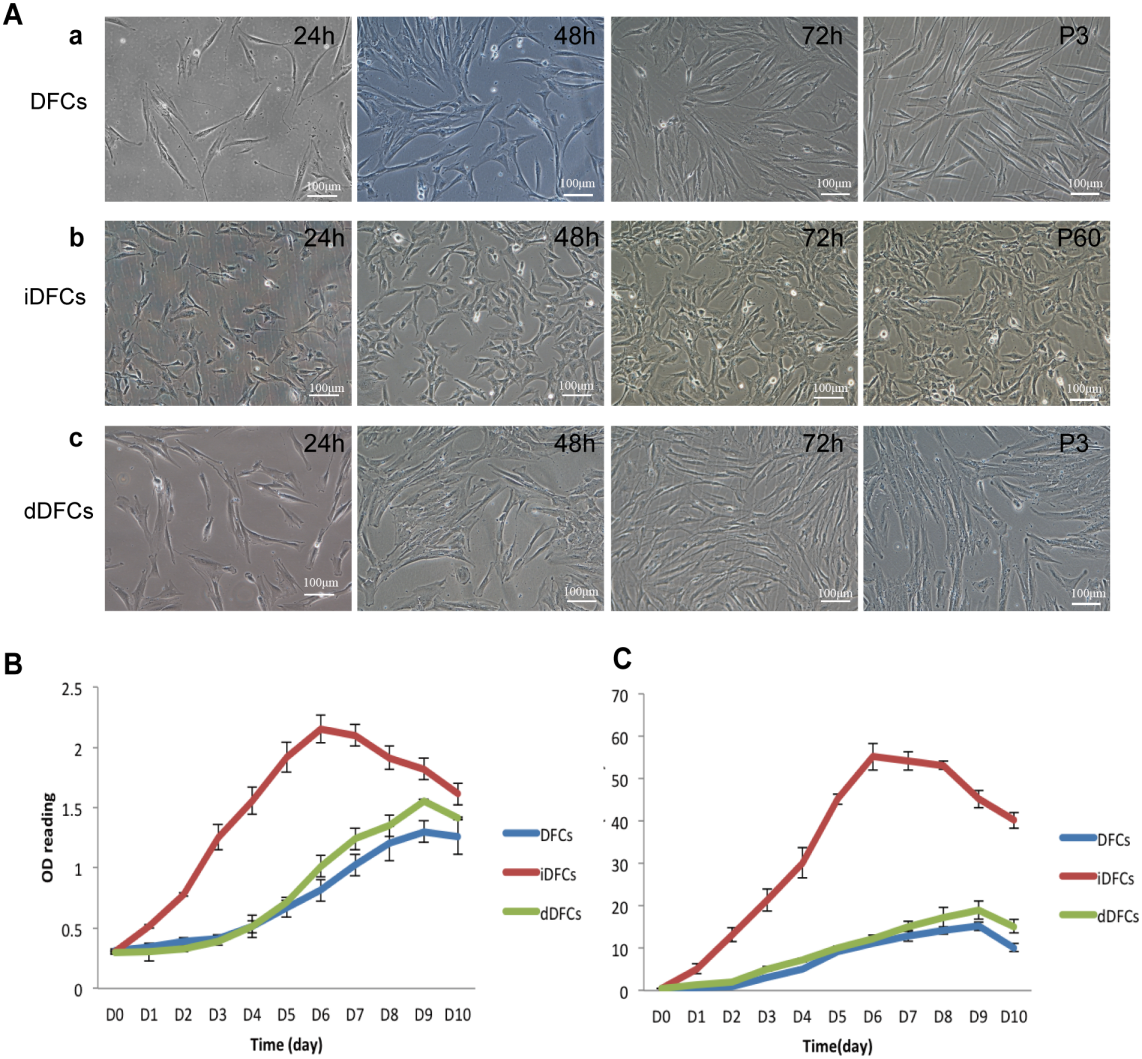
Morphology and cell proliferation of three types of DFCs. (A) Morphology of primary dental follicle cells (DFCs), immortalized dental follicle cells (iDFCs) and deimmortalized dental follicle cells (dDFCs). Primary DFCs and deimmortalized DFCs were seeded at 20% confluence and cultured for three passages (*P3*)(a and c). iDFCs were seeded at 20% confluence and cultured for 60 passages (*P60*) (b). (B) Cell proliferation assessed with CCK8 assay. (C) Cell growth curve.

The immortalized DFCs (iDFCs) with SV40 T-Ag gene could maintained their fibroblast-like morphology (Fig 3A, panel b) even at passage 60 and grew more rapidly (Fig 3B and 3C), compared to DFCs. The dDFCs lost high proliferation rate and the growth rate was similar to primary DFCs, which was proved both by the cell counting method (Fig 3C) and CCK-8 test (Fig 3B).

Taken together, the results showed that iDFCs can be maintained in culture and display higher proliferation rate than primary DFCs, and iDFCs could be reversed back to normal primary DFCs in cell proliferation pattern by deimmortalization.

### iDFCs and dDFCs express DFCs markers

**Fig 4:**
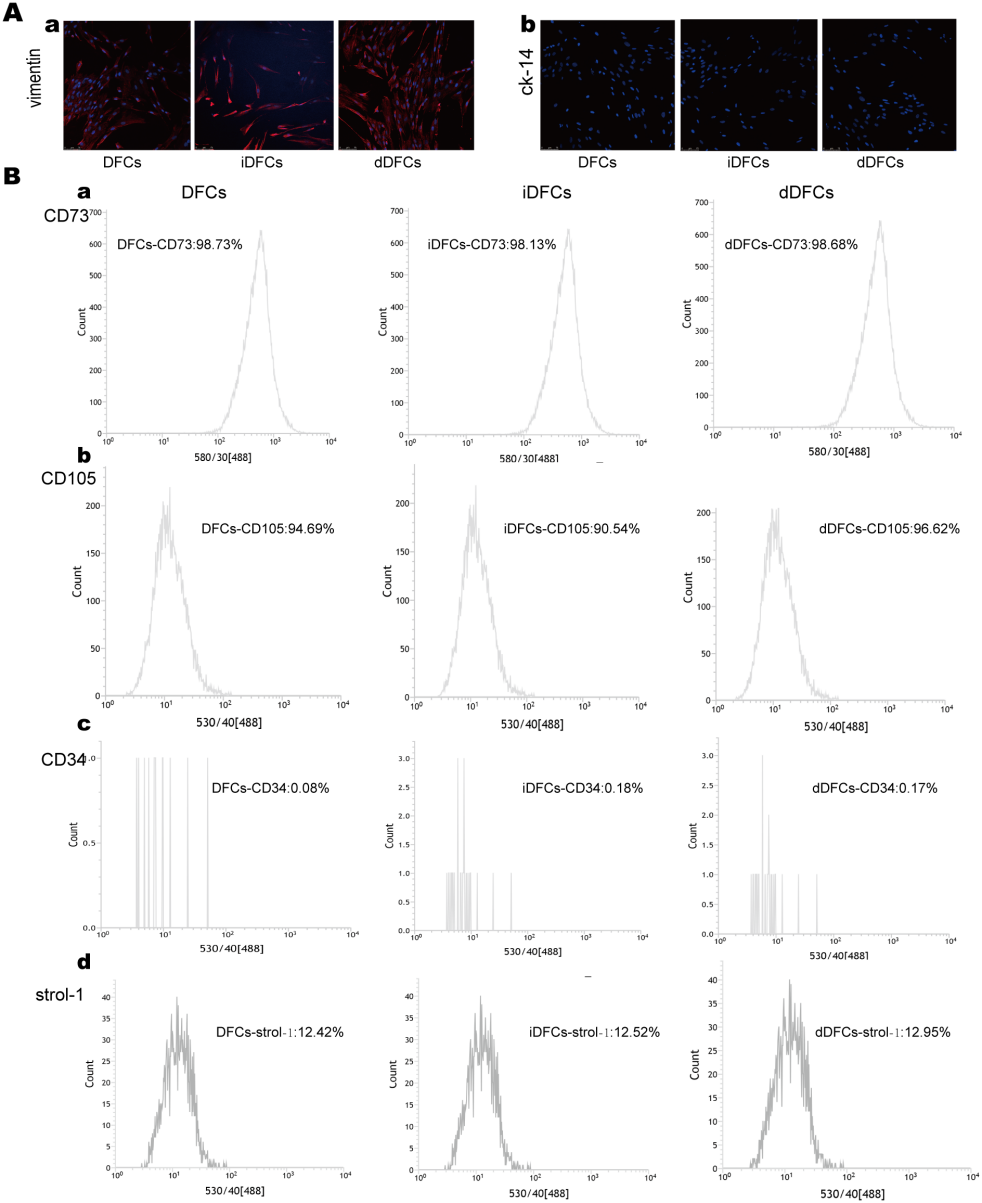
The DFCs, iDFCs and dDFCs express mesenchymal stem cell surface markers. (A) iDFCs and dDFCs possess DFCs properties. iDFCs and dDFCs are both vimentin positive and ck14 negative just the same as DFCs. (B) DFCs, iDFCs and dDFCs express mesenchymal stem cell surface markers, i.e. CD73 (a) and CD105 (b), but lack the expression of CD34(c). The percentage of strol-1 positive cells is about 13%(d).

Both iDFCs and dDFCs are vimentin positive and CK14 negative, which was similar to DFCs (Fig 4A). Above 90% of iDFCs, dDFCs were CD105, CD73 positive but CD34 negative as the same as primary DFCs (Fig 4B, panel a-c). The percentage of strol-1 positive cells in iDFCs and dDFCs are about 13%, which is also similar to primary DFCs. (Fig 4B, panel d).

### iDFCs maintain high telomerase activity

**Fig 5:**
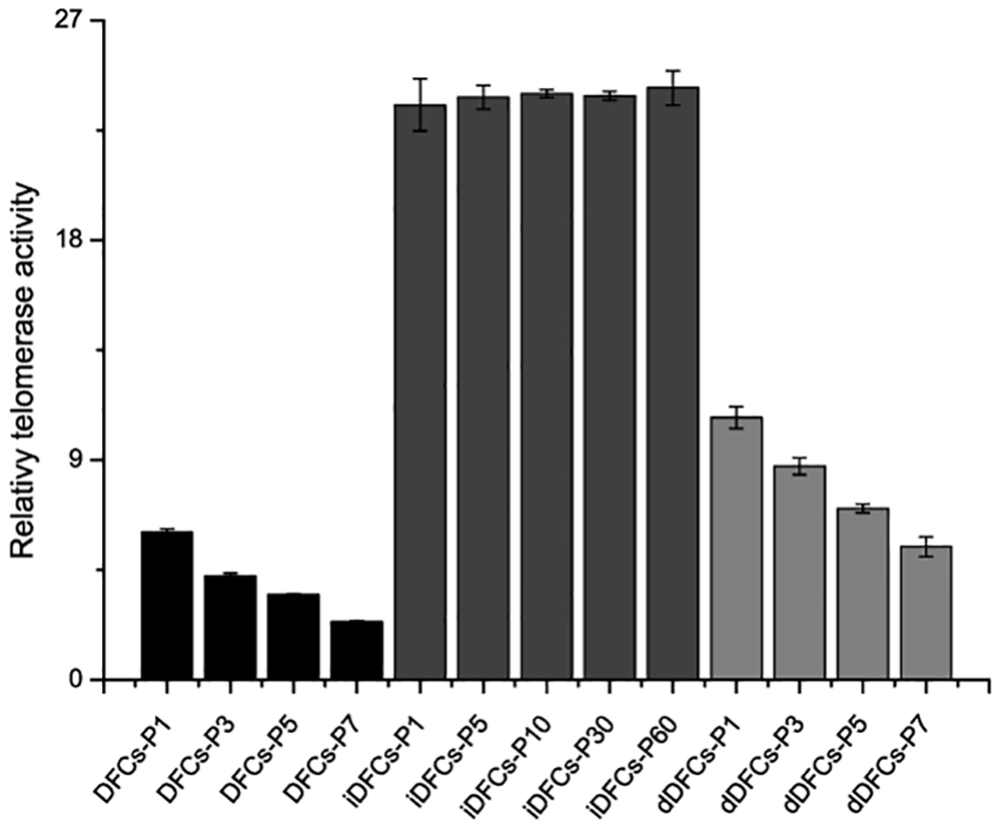
Telomerase activity of three types of DFCs. iDFCs maintain high telomerase activity while the telomerase activity of dDFCs is similar to primary DFCs. Telomerase activity of primary DFCs and dDFCs was tested at *p1*, *p3*, *p5* and *p7*. Telomerase activity of iDFCs was tested at *p1*, *p5*, *p10*, *p30*, and *p60*. The telomerase activity of cells was measured by telomeric repeat amplification protocol.

Telomerase activity of iDFCs are significantly increased and maintained at a high level till passage 60, while the telomerase activity of primary DFCs was relatively low and decreased with every passage due to possible nondirectional differentiation. After SV40 T-Ag was removed to deimmortalize the cells, the telomerase activity reduced to its original level before immortalization and decreased with passages as the same as primary DFCS (Fig 5).

### Differentiation of iDFCs and dDFCs

#### Osteogenic differentiation

**Fig 6:**
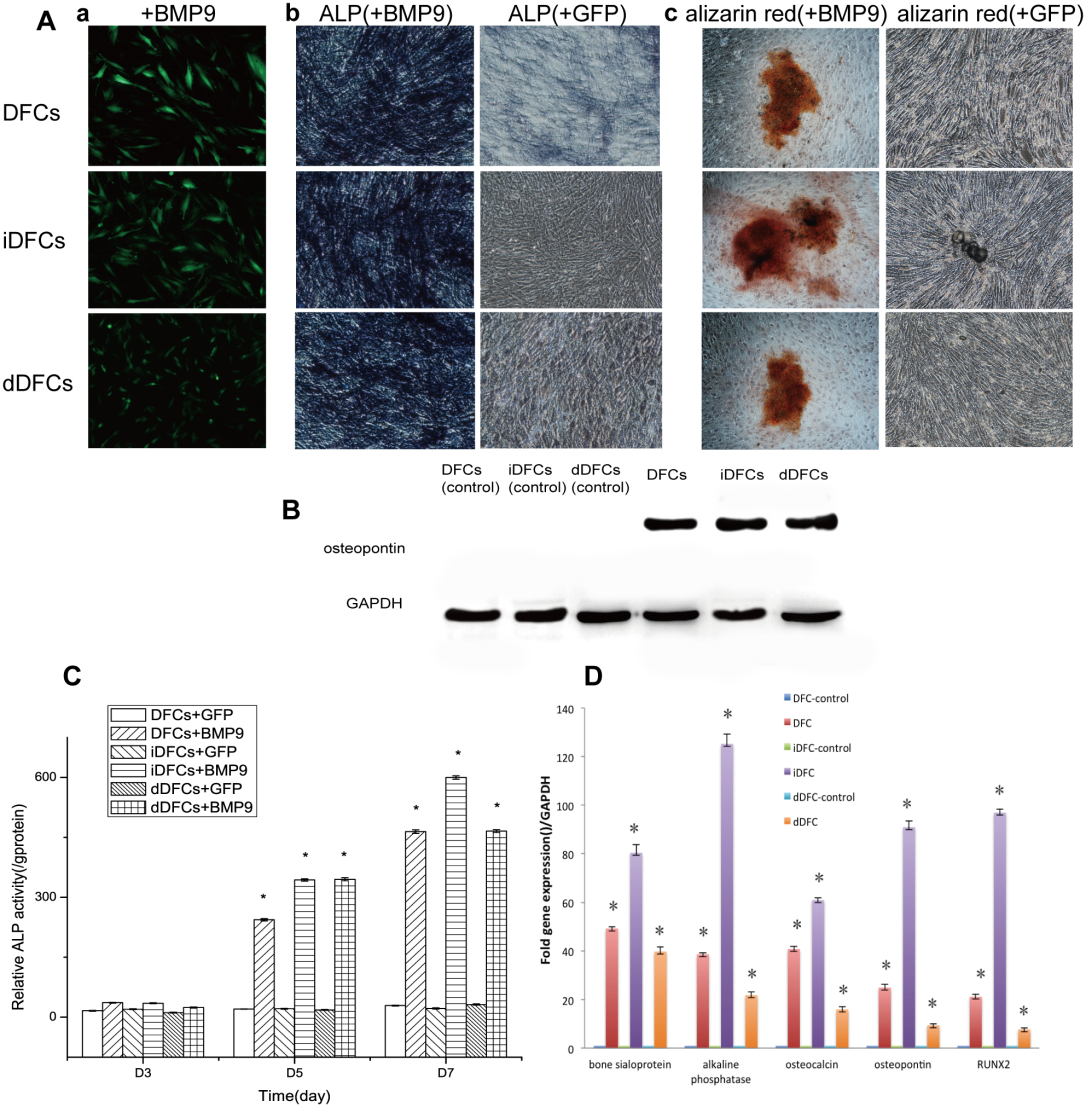
Osteogenic differentiation of DFCs, iDFCs and dDFCs. (A) Induction of alkaline phosphatase (ALP), the early stage of osteogenic differentiation marker and alizarin red, the late matrix mineralization marker in DFCs, iDFCs and dDFCs after osteogenic differentiation. Subconfluent DFCs, iDFCs and dDFCs were infected by adenovirus AdBMP9 (a) or AdGFP (data unshown). ALP activity of cells was stained on day 7 and measured quantitatively at days 3, 5 and 7 (c). (B) The expression of osteopontin after osteogenic Induction of DFCs, iDFCs and dDFCs. Cells were transducted by AdBMP9 (a) or AdGFP. Anti-GAPDH Western blotting ensures the same amount of samples loaded. Samples infected by AdGFP didn’t express osteopontin. (D) Expression of osteogenic lineage-specific genes in DFCs, iDFCs and dDFCs after being induced by BMP9. Cells were transducted with AdBMP9 or AdGFP as negative control. The assays were done in three experiments. Note: *p < 0.05.

After transduced with BMP9 adenoviral vector, DFCs, iDFCs and dDFCs all expressed similar levels of osteopontin protein marker in Western blot (Fig 6B) and ALP protein makers (Fig 6A, panel b) and the genes related to osteogenic differentiation were significantly up-regulated compared with control groups which were infected with GFP adenoviral vector alone (Fig 6D). The AdBMP9-infected DFCs, iDFCs and DFCs were further proved to develop late phase of osteogenic differentiation as verified by Alizarin Red S staining (Fig 6A, panel c).

#### Adipogenic differentiation

**Fig 7:**
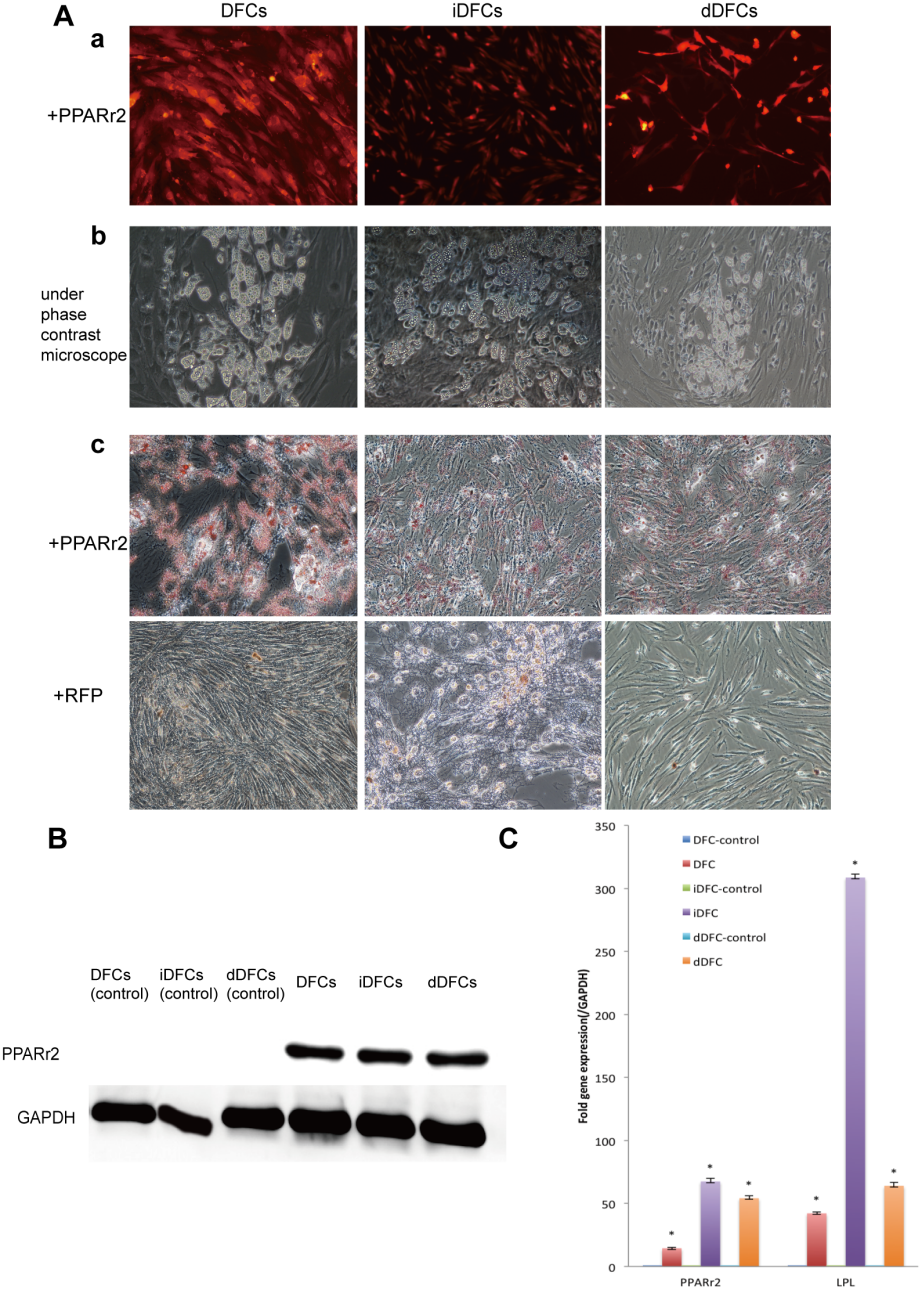
Adipogenic differentiation of DFCs, iDFCs and dDFCs. (A) DFCs, iDFCs and dDFCs can be induced to form adipocyte-like cells. Subconfluent DFCs, iDFCs and dDFCs were infected by adenovirus Ad PPARγ2 (a). or AdRFP (data unshown). 5 days after adipogenic induction, lipid droplets can be seen clearly under phase contrast microscope (b). Lipid droplets can be stain by oil red as compared to the control groups infected by AdRFP(c). (B) The expression of PPARγ2 after adipogenic differentiation of DFCs, iDFCs and dDFCs. Subconfluent cells were transducted by AdPPARγ2 or AdRFP as negative control. On day 5, western blotting was performed using anti- PPARγ2 antibody. Anti-GAPDH Western blotting ensures the same amount of samples loaded. Samples infected by AdRFP didn’t express PPARγ2. (D) Expression of adipogenic lineage-specific genes in DFCs, iDFCs and dDFCs stimulated by PPARγ2. The assays were done in three experiments. Note: *p < 0.05.

Following infected with Ad PPARγ2, about 70% of DFCs, iDFCs and dDFCs expressed PPARγ2 and LPL gene assessed by PCR, Oil Red-O staining positive (Fig 7A, panel c and Fig 7C), PPARγ2 protein maker in Western blot (Fig 7B) respectively.

#### Chondrogenic differentiation

**Fig 8:**
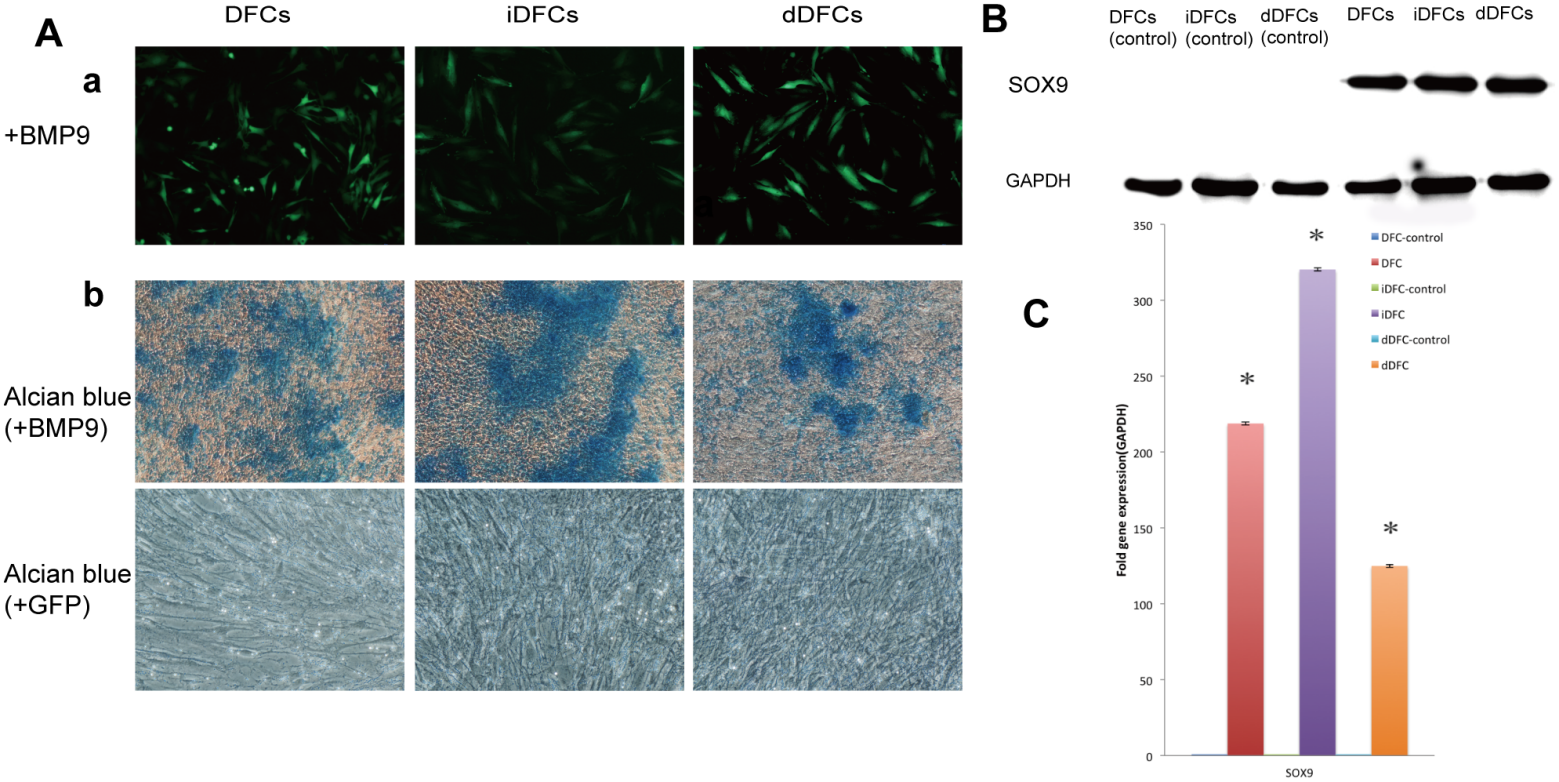
Chondrogenic differentiation of DFCs, iDFCs and dDFCs. (A) Induction of Alcian blue in DFCs, iDFCs and dDFCs after chondrogenic induction. Subconfluent DFCs, iDFCs and dDFCs were infected by adenovirus AdBMP9 (a) or AdGFP (data unshown). Alcian blue staining was done after the cells were chondrogenic differentiated for one week (b). (B) The expression of SOX9 after chondrogenic differentiation of DFCs, iDFCs and dDFCs. Subconfluent cells were infected by AdBMP9 (a) or AdGFP. On day 14, cells were lysed, and then subjected to Western blotting using anti- SOX9 antibody. Anti-GAPDH Western blotting ensures equal loading of the samples. Samples infected by AdGFP didn’t express SOX9. (C) Expression of chondrogenic lineage-specific regulators in DFCs, iDFCs and dDFCs stimulated by BMP9. Cells were transfected with AdBMP9 or AdGFP. The assays were done in three experiment. Note: *p < 0.05.

After transduced with BMP9 adenoviral vector (Fig 8A, panel a), DFCs, iDFCs and dDFCs displayed Alcian Blue staining positive (Fig 8A, panel b), expressed SOX9 genes (Fig 8C) in PCR and SOX9 protein maker (Fig 8B) in Western blot related to chondrogenic differentiation, compared with control groups infected with GFP adenoviral vector alone.

Taking these results together, iDFCs and dDFCs are multi-potent as DFCs and are able to differentiate into adipogenic, chondrogenic and osteogenic cells. And we also find that iDFCs expressed higher levels of related gene when differentiated compared to DFCs or dDFCs.

## Discussion

Dental follicle is the connective tissue wrapped around the developing dental germ before tooth erupted. DFCs, originated from dental follicle, have attracted much attention because they play an essential role in the development of alveolar bone cementum and periodontal ligament. [37,38]. Substantial studies proved that DFCs possess stem cell-like properties evidenced by their potential to be induced to become osteogenic, adipogenic, chondrogenic cells both *in vitro* and *in vivo* [3].

However, isolation of primary DFCs is labor-intensive and time-consuming and DFCs are sentenced to cellular senescence, that is to say the cells can only proliferate *in vitro* for a limited number of passages. Therefore, how to immortalize the cells to prevent their cellular senescence is essential for cell culture and amplification. Basically, three approaches are used to develop immortalized cells, including spontaneous random mutagenesis after serial passages of primary cells, overexpression of oncogenes and Inhibition of tumor suppressor gens. The BALB/3T3 cell line[39] is establish through serial passage while most of the other immortalized cell lines are established by overexpression of oncogenes, including Bmi-1, TERT [37], SV40 T-Ag, E1A E1B, and HPV 16 E6/E7, etc, or inactivativation of tumor suppressor genes including retinoblastoma,p53 and p16INK.

Stable expression of SV40 T-Ag by using a retroviral vector-based reversible immortalization system expressing SV40 T-Ag has been used previously to immortalize many kinds of primary cells, including mouse hepatic cells, mouse embryonic fibroblasts mouse cardiomyogenic cells, and mouse melanoblastic cells [17,19–23]. However, immortalization efficiency of these cells was low, because the viral titters of retrovirus was low when large cargo size is transducted. and the mutations of the host DNA after the infection of retrovirus is also of great concern. So we intend to utilize the *piggyBac* transposon-mediated system with overexpression of SV40 T-Ag to immortalize DFCs, and to investigate whether the multi-differentiation potential of DFCs could be retained or not.

The *piggyBac* transposon system is a mobile genetic unite that can effectively catalyze integration and excision of transgenes in human cells between vectors and host genome through a direct "cut and paste" mechanism called transposition [27,40,41]. It has been proved to be a more effective transposon system in different types of mammalian cells compared with other transposons such as Tol2 and Sleeping Beauty11 [42], which makes it the ideal DNA transposon for gene delivery system [40]. It has great advantages over retroviral system [24,43]. First of all, it can integrate foreign fragments up to 100 kb in human and mouse embryonic stem cells[44,45]. Secondly, it can catalyze integrate of single copy of foreign DNA element at multiple locations with high frequency within the host genome [46]. Moreover, it has been reported the *piggyBac* transposon doesn’t integrate near active genes or cancer-related genes, and thus less likely to triger detrimental mutations[47]. Lastly, *piggyBac* transposase can be used to remove the inserted *piggyBac* vector from host genomic DNA footprint-free.

So, we intend to investigate whether *piggyBac* transposon-mediated overexpression of SV40 T-Ag can establish immortalized DFCs effectively. We demonstrated the *piggyBac* system can effectively immortalize DFCs by introducing SV40 T-Ag into host cells through the activation of telomerase activity, and the resulting iDFCs proliferate much faster than DFCs, and the deimmortalized DFCs are settled after the immortalization gene SV40 T-Ag were removed by FLP recombinase. The AdpBase that express *piggyBac* transposase we use in this experiment will integrate and remove pMPH86 plasmid with equal efficiency, so we designed a FRT cite and use the FLP system to remove the SV40 Large T antigen effectively. But the innovated excision-dominate form of *piggyBac* transposase have already been reported [48,49].

As previously reported, there are no specific surface markers for mesenchymal stem cells, while the commonly accepted minimal phenotypic criteria is expression of CD73 and CD105, and lack the expression of CD34 [50]. And also strol-1 is considered to be an important marker for mesenchymal stem cells. In our experiment, more than 90% percent of the immortalized DFCs and the deimmortalized DFCs are CD73 and CD105 positive while the expression of CD34 is very low (less than 1%). The ratio of strol-1 positive cells are alike in DFCs,iDFCs and dDFCs. Thus, we can conclude the immortalized and deimmortalized DFCs didn’t lose the mesenchymal stem cell surface marker. We also proved the efficiency of *piggyBac* transposon system is much higher than the previously used retrovirus system.

Given the fact that mesenchymal stem cells are multi-potent cells that possess the potential to be induced into chondrocytes, osteoblasts and adipocytes [1–5], we tested whether iDFCs and dDFCs were capable of differentiation into these cell lineages. As previously proved, BMP9 has been shown to greatly improve the osteogenic and chondrogenic differentiation capacity of cells [29–33]. In our study, upon BMP9 stimulation, iDFCs and dDFCs can undergo osteogenic and chondrogenic differentiation, which is proved by RT-PCR and western blot. And while the iDFC and dDFCs are transfected by AdPPARγ2, the cells can differentiate into adipocyte-like cells, which can be stained by oil-red and confirmed by RT-PCR and western blot results.

Although the immortalized DFCs proliferated faster than the primary DFCs, when the SV40 T-Ag are removed, the dDFCs exhibit a more similar proliferation rate to DFCs, and the telomerase activity are down-regulated also, which is an indirect evidence that the iDFCs are not tumorigenic and dDFCs encompass the same proliferate capacity as primary DFCs. Thus, the immortalization method can be used as a safe method to amplify DFCs without sacrificing their multi-potent differentiation capacity or causing any potential tumorigenic effect.

In summary, primary DFCs are amplified with higher cell prolifereation after transfected with SV40 T-Ag gene via *piggyBac* transpons-mediated reversible immortalization System. DFCs can be safely revised back to primary cell types in cell proliferation and differentiation capacity, which makes their potential use in research and clinic much reliable.
